# Clinical value of whole-body magnetic resonance imaging in health screening of general adult population

**DOI:** 10.2478/raon-2014-0031

**Published:** 2015-03-03

**Authors:** David Laszlo Tarnoki, Adam Domonkos Tarnoki, Antje Richter, Kinga Karlinger, Viktor Berczi, Dirk Pickuth

**Affiliations:** 1 Department of Diagnostic and Interventional Radiology, Caritasklinikum Saarbrücken St. Theresia, Academic Teaching Hospital of Saarland University, Saarbrücken, Germany; 2 Department of Radiology and Oncotherapy, Semmelweis University School of Medicine, Budapest, Hungary

**Keywords:** angiography, incidentaloma, atherosclerosis, high field magnet, diffusion weighted imaging

## Abstract

**Background:**

Whole-body magnetic resonance imaging (WB-MRI) and angiography (WB-MRA) has become increasingly popular in population-based research. We evaluated retrospectively the frequency of potentially relevant incidental findings throughout the body.

**Materials and methods:**

22 highly health-conscious managers (18 men, mean age 47±9 years) underwent WB-MRI and WB-MRA between March 2012 and September 2013 on a Discovery MR750w wide bore 3 Tesla device (GE Healthcare) using T1 weighted, short tau inversion recovery (STIR) and diffusion weighted imaging (DWI) acquisitions according to a standardized protocol.

**Results:**

A suspicious (pararectal) malignancy was detected in one patient which was confirmed by an endorectal sonography. Incidental findings were described in 20 subjects, including hydrocele (11 patients), benign bony lesion (7 patients) and non-specific lymph nodes (5 patients). Further investigations were recommended in 68% (ultrasound: 36%, computed tomography: 28%, mammography: 9%, additional MRI: 9%). WB-MRA were negative in 16 subjects. Vascular normal variations were reported in 23%, and a 40% left proximal common carotid artery stenosis were described in one subject.

**Conclusions:**

WB-MRI and MRA lead to the detection of clinically relevant diseases and unexpected findings in a cohort of healthy adults that require further imaging or surveillance in 68%. WB-MR imaging may play a paramount role in health screening, especially in the future generation of (epi)genetic based screening of malignant and atherosclerotic disorders. Our study is the first which involved a highly selected patient group using a high field 3-T wide bore magnet system with T1, STIR, MRA and whole-body DWI acquisitions as well.

## Introduction

Whole-body magnetic resonance imaging (WB-MRI) has become increasingly popular in the recent decade due to its high soft tissue spatial resolution, multiplanarity, lack of ionising radiation, low incidence of nephrotoxicity caused by contrast agents, as well as high sensitivity and specificity in the detection of vascular and malignant diseases.^1^,^2^ Due to financial reasons and limited availability, WB-MRI enables an early diagnosis mainly in defined groups of subjects who do not show symptoms yet. The most common diseases in elderly population include malignant tumours and cardiovascular diseases.^3^ WB-MRI is capable of detecting a wide range of malignant diseases; such as bronchial carcinoma, hepatic malignancies, renal carcinoma, colonic cancer, lymphoma, and also rare malignancies such as bone or soft tissue tumours.^4^

With wider availability of WB-MRI, increased number of incidental findings of potential clinical relevance (36%) have been reported.^5^ Only a limited number of studies on screening with 1.5-T WB-MRI is available in the literature, often restricted to a single organ system.^5^ However, the prevalence of incidental findings has not yet been described on a 3-Tesla (T) wide bore device. Furthermore, no screening studies including both whole-body diffusion weighted imaging (DWI) and WB-MRA have been performed.

Our aim was to retrospectively analyze the frequencies of potentially relevant incidental findings throughout the body, especially in view of those that require further medical evaluation.

## Materials and methods

**FIGURE 1.**
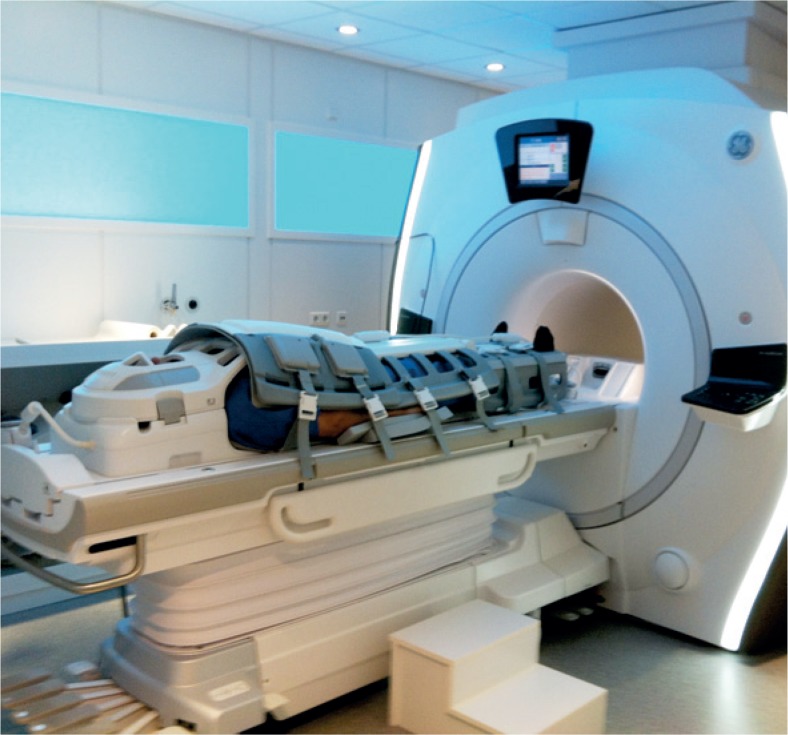
Whole-body MRI set-up of coils (head, chest, abdomen and extremity coils) in a wide bore magnet device.

**FIGURE 2. A–C:**
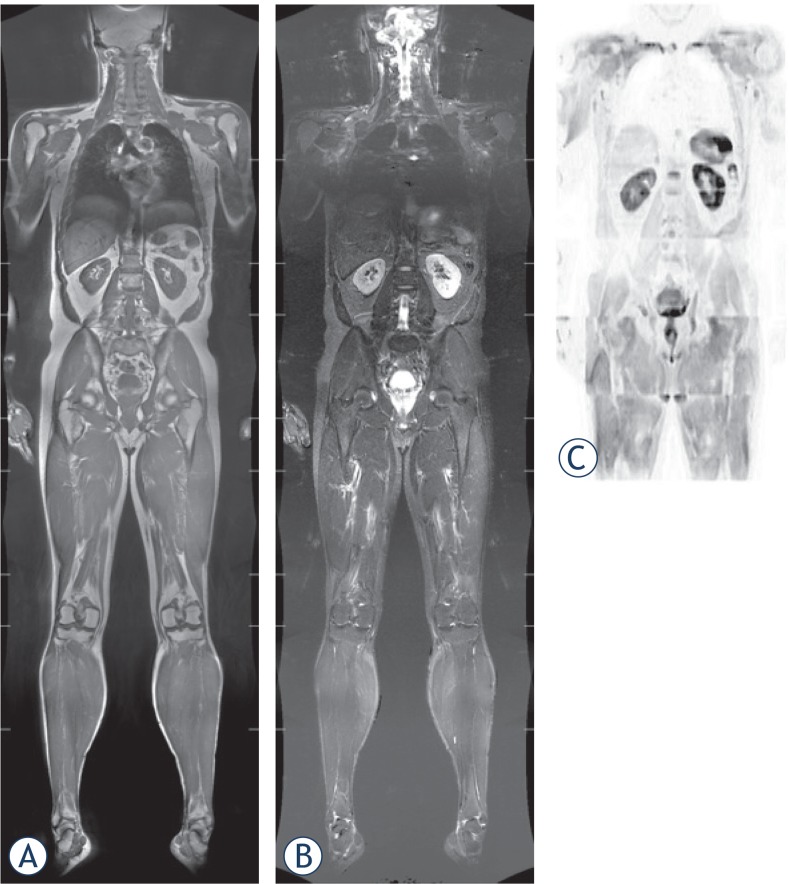
Whole-body MRI acquired on a dedicated whole-body MRI system and matrix coils in asymptomatic, smoker male 46-year-old patient, coronal **(A)** T1, **(B)** STIR and **(C)** DWI images.

We carried out a retrospective analysis of healthy adults (mainly managers, lawyers, accountants, chief executive officers, company directors) with extreme health consciousness who underwent whole-body MRI and MRA at the Institute of Diagnostic and Interventional Radiology, Caritasklinikum Saarbrücken St. Theresia, Germany between March 2012 and September 2013. Subjects were referred to WB-MRI scan by a family doctor, their company or were self referrals. The healthy adults completed a comprehensive questionnaire including their current symptoms, previous clinical findings, operations, and risk factors. MR studies were acquired on a Discovery MR750w 3 Tesla wide bore device (General Electric Healthcare, GE, Milwaukee, USA; 70 cm wide bore magnet) using T1 weighted (fast Spin Echo technique with a slice thickness of 5 mm), short tau inversion recovery (STIR), and DWI sequences according to a standardized protocol (Figure 1, 2). Depending upon the height of the patient, 6 or 7 slabs were acquired in a slab-by-slab-technique with no continuous table movement. The WB-MRI protocol was identical for all participants and included a plain WB-MRI and detailed examination of head, neck, chest, abdomen, pelvis, spine and extremities. A rolling platform with extended field of view allowed whole-body examinations with a table range of more than 200 cm, several dozen simultaneous receiver channels, and multiple plugs for attaching several RF coils concurrently enabling the individual to be covered with coils from “head to toe”. The high number of coils allowed for parallel imaging which speeded up the data acquisition.

**FIGURE 3.**
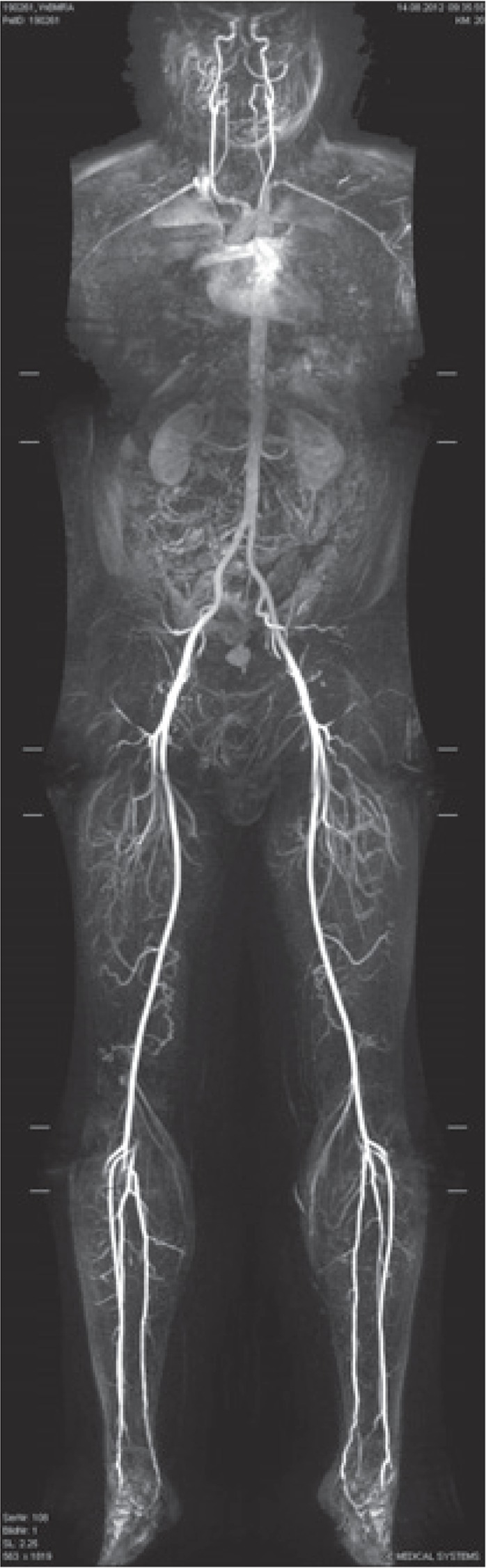
Whole-body MRA acquired on a dedicated whole-body MRI system and matrix coils with a single injection of contrast agent in an asymptomatic male 52-year-old patient, coronal reconstruction, pasted image.

MR angiography (using 3D technique) was performed by the administration of 0.45 ml/kg body weight gadolinium contrast agent (0.5 mmol/ml gadoterate meglumine, Dotarem, Guerbet, Roissy, France), and was automatically injected at a flow rate of 1.2 ml/s in the first and 0.6 ml/s in the second phase (Figure 3). The patient was placed in the supine position, and the phases were acquired using respiratory gating.

Findings and anatomical variants were documented in a standardized reading protocol. Picture archiving and communication system (AGFA IMPAX and KIS-RIS ORBIS, AGFA Healthcare, Mortsel, Belgium) were used for image storing. In order to ensure the best possible quality and to minimize the inter-reader variability, three readers reported the whole set of images: first-line reading was performed by a resident in radiology (2–4 years’ experience), followed by two senior radiologists including the head of the department. The three readers evaluated the studies independently from each other. Then the results were discussed in consensus. Finally, opinion, diagnoses/differential diagnoses and recommendation were evaluated together. The evaluation of images were carried out retrospectively by reviewing the reports and images of the patients. Findings were classified as normal, insignificant (abnormalities without well-defined diagnostic and therapeutic consequences according to existing guidelines and best practice recommendations), potentially significant (abnormalities potentially needing further medical evaluation or follow-up), and significant (findings that require further medical evaluation and immediate referral). Statistical analysis was performed by Microsoft Excel. The investigators followed the Helsinki Declarations and the European Council Convention on Protection of Human Rights in Bio-Medicine.

## Results

### Subject characteristics

Twenty-two healthy adults (18 men, age 47±9 years, mean±standard deviation) who underwent whole-body MRI and MRA imaging between March 2012 and September 2013 were included in the study. The WB-MR scans were analyzed retrospectively. The mean body mass index of the subjects was 25.2 kg/m^2^. Fourteen subjects were completely asymptomatic. Nine subjects had a history of allergy (*e.g*., drug, animals, pollens). Nineteen subjects were never smokers, two reported a previous smoking history and one was active smoker. Subjects reported never, occasional, and regular sport activity in 5%, 10% and 85%, respectively. Four-fifth of the subjects had current symptoms, previous symptoms/surgeries.

### WB-MRI findings

**FIGURE 4. A–C:**
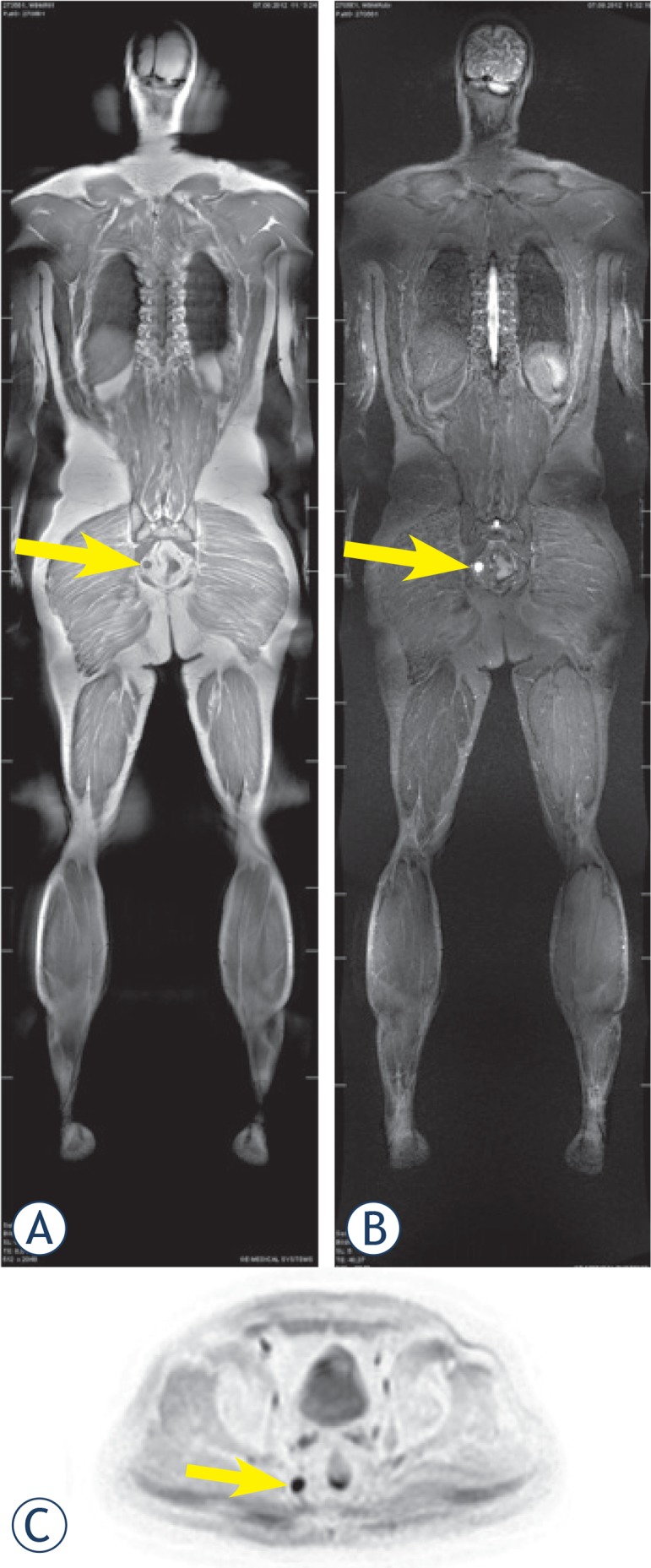
Whole-body MR image of a 52-year-old male patient with history of sleep disorder, night sweats, hypercholesterinaemia and previous smoking. Note the right pararectal 16×14 mm mass (arrows) on the coronal **(A)** T1 (hypointense signal) and **(B)** STIR images (hyperintense signal); **(C)** diffusion weighted (DWI) axial image indicating a restricted diffusion. Inverted DWI image is similar to a PET image.

**FIGURE 5.**
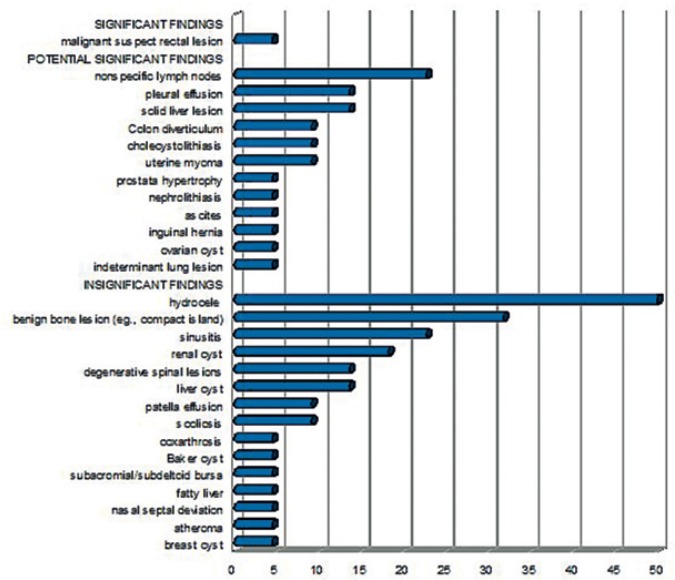
Prevalence of WB-MRI findings in all patients (%).

A suspicious (pararectal) malignancy was detected in one patient (Figure 4). Two patients had negative MR reports, whereas incidental findings were described in 20 subjects. The findings are shown in Figure 5. Hydrocele was the most common incidental finding (11 patients; 11 of 18 men), followed by a benign bony lesion in 7 patients. Incidental findings would have needed diagnostic workup at an urologist (17 lesions), rheumatologist (15 lesions), internist (13 lesions), otorhinolaryngologist (6 lesions), pulmonologist (6 lesions), surgeon (5 lesions), gynecologist (4 lesions), and dermatologist (1 lesion). Further investigations were recommended in 68% of subjects including eight sonographies (2/3 abdominal), five chest computed tomographies (CT), one pelvic CT, two mammographies and two additional MRIs. In case of the suspicious pararectal malignancy, biopsy was recommended. The patient had an endorectal sonography which confirmed the presence of a highly suspicious mass, probably a lymph node. A rectoscopy/colonoscopy was planned, however the patient moved to another city and the further diagnostic/therapeutic workup is unknown yet.

### WB-MRA findings

**FIGURE 6.**
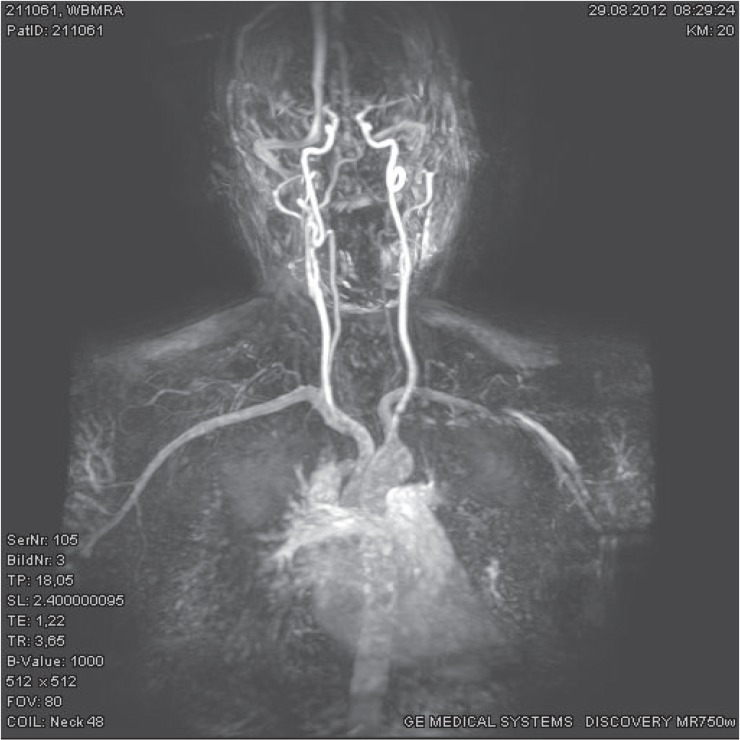
Coronal reconstruction of whole-body MRA in a 52-year-old male patient with history of appendectomy and previous smoking. Note the 2 mm long, approximately 40% concentric stenosis of the left common carotid artery 3 cm distal to the aortic arch (arrow). Please note the left vertebral hypoplasia, kinking of the midcervical portion of the right internal carotid artery and the coiling of the distal cervical portion of the left internal cerebral artery.

WB-MRA was negative in 16 subjects. Vascular normal variations (*e.g*. irregular caliber of the vertebral artery, polar renal artery, stronger posterior communicant artery) were reported in five subjects, and a non-significant stenosis was described in one subject (Figure 6). A further subject had a possible right subclavian stenosis which might be confounded by motion artifact.

## Discussion

To the best of our knowledge, this is the first study to investigate the clinical value of whole-body MRI and MRA in a highly selected group of extremely health conscious general adult population on a 3-T basis using a wide bore magnet including WB-DWI acquisitions. We demonstrated a potentially malignant lesion detection rate of 4.5% in our cohort and a high number of incidental findings (91%) requiring further radiological investigations in 68% of individuals. WB-MRA demonstrated normal vascular variations in 23% of subjects and a non-significant left proximal common carotid artery stenosis in 4.5%.

The patient, who had a suspicious (pararectal) malignant lesion underwent a pelvis CT, which found a 16 × 14 mm large mass with central discrete hypodensity in the right pararectal fat tissue and a thickened mesorectal fascia. Tumor markers were in the normal range, and colonoscopy reported sigma polyps without dysplasia (no suspicous colon carcinoma). The patient did not show up in the follow-ups later.

Only few studies performed on a lower magnetic field (usually 1.5 T) have reported WB-MRI screening results in the past years.^4^,^5^ Gohde *et al.* reported lower rates of clinically significant incidental findings compared to our results in general (*e.g.* peripheral arterial stenoses in 2%, significant incidental findings 5–9%), and demonstrated only one malignancy (a renal cell carcinoma).^6^ Studies performed on ‘healthy’ employees reported only few unknown vascular pathologies, usually below 5% (*e.g.* silent myocardial infarction, cerebral infarctions, significant carotid/renal artery stenoses).^7^–^9^ Some population-based studies indicated similar prevalence, for example the Uppsala PIVUS study (performed in 306 70-year-old men) demonstrated significant carotid/renal artery stenoses in 1.5–1.8%, and abdominal aortic aneurysms in 2%.^10^ A study of 2536 healthy young men using axial brain MRI reported brain incidental findings with frequency of 0.47–1.7%.^11^ We suspect that the higher prevalence of incidental findings in our study can be referred to the higher magnetic field and the technical novelties in MRI, such as extended body coverage, a rolling platform with extended large field of view and a high number of simultaneous signal receiver channels, and multiple plugs for attaching several RF coils concurrently – allowing “parallel imaging”.^1^,^12^ In our study, homogeneity of the main magnetic field B0 of 3 T bore magnet with the largest bore diameter available on the market, higher signal to noise ratio, better image resolution, shorter acquisition time, and more patient comfort can further increase and facilitate the specificity and patient compliance in contrast to the 1.5 T MRI systems. Spatial resolution data of our MR acquisitions are shown in Table 1.

In our cohort, hydrocele was the most common incidental finding (61% of men). The cause is unknown, however, it might be a marker of physical trauma, infection, or tumor as well. There are approximately 26 million cases of hydrocele worldwide.^13^ Hydrocele is more common on the right side and it is usually bilateral in elderly patients.^14^

The second most common incidental finding was the benign bony lesion in 32% of the individuals, most commonly bone islands (these lesions appeared to be benign but were unspecific, maybe just inhomogeneous bone marrow). These small (majority of lesions measure from 0.1 to 2.0 cm in greatest diameter), asymptomatic lesions can be found in most parts of the skeleton with a preference for the pelvis, femur, ribs and other long bones (usually at the ends of tubular bones).^15^ Most of these bone islands do not require treatment after the diagnosis is established.

Due to atherosclerosis as the number one in morbidity and mortality in developed countries and its high prevalence, there is an increasing need to detect the most threatening manifestations of vascular disease well in advance.^16^ WB-MRA is a promising technique providing the depiction of the arterial system from “head to toe” (except the coronaries) in less than 45 minutes, visualizing macroscopic changes in the arterial system and potential organ damage (*e.g*. cerebral microangiopathy, stroke, myocardial infarction) with high accuracy of up to 95% concerning relevant stenosis.^1^,^17^ In our sample, WB-MRA was negative in 73%, and vascular normal variations and a non-significant stenosis were described. Our findings are comparable with studies performed in asymptomatic patients for cardiovascular diseases where the prevalence for vascular (< 3 T) MR findings were relatively low.^4^,^9^,^10^ However, in high-risk groups, screening studies have revealed many previously unknown vascular pathologies, which in part had been over-seen by common clinical examinations and tests.^4^ Our most commonly reported vascular normal variation was the asymmetry of the vertebral arteries (VAs). The asymmetry of the VAs might be due to hypoplasia which is very common and can be identified on MRI, its prevalence is unknown.^18^ A Swiss study reported that vertebral artery hypoplasia is more common on the right side.^19^ Hypertension or hyperlipidemia are hypothesized to be in the background; furthermore, vertebral artery hypoplasia may contribute to a higher risk for posterior circulation stroke.^19^

WB-MRI can be performed by the application of various sequences. T1-weighted images after contrast agent application can depict lesions in parenchymal organs and bone and soft tissues due to higher spatial resolution.^1^,^20^ STIR can visualize vertebral metastases and bone marrow infiltration with high sensitivity.^21^ Due to these benefits, these sequences were also acquired in our study in accordance with the literature. However, to the best of our knowledge, DWI has never been applied in a WB-MRI screening study. Advantage of adding a DWI sequence to this study has the depiction of areas of restricted diffusion which allows the better visualization of areas of high cellularity, *i.e*. malignancies.

The ideal screening technique must be both sensitive and specific, widely available, cost-effective, reader independent, and without harmful side effects. The diagnostic test must be standardized implying a low number of false results.^1^ In addition, criteria for a screening programme (either Wilson and Jungner, or adapted WHO) should be met. MR is likely to meet these criteria especially in diseases which are ideal for screening, including colorectal cancer and cardiovascular disease, as demonstrated with examples in our study.^1^,^22^,^23^ MR angiography has also been shown to be equivalently effective in demonstrating vascular abnormalities compared to invasive techniques.^1^,^24^ In our study, further investigations were recommended in case of 15 subjects (68%), mainly abdominal sonographies and chest CTs. The incidental findings were mainly related to the fields of urology, rheumatology and internal medicine in almost two-thirds of the cases. The corresponding screening costs are also determined by these indirect costs related to these subsequent and follow-up tests beyond the direct costs of the screening test itself.^1^

The strengths of our study include the highly selected patient group, the advantages of high field 3-T wide bore magnet system and the use of whole-body DWI acquisitions. Second, the investigated healthy adults represent a small proportion of the population at risk, and the prevalence of malignancies and cardiovascular (atherosclerotic) lesions are likely to occur in the higher-risk group with lower socioeconomic status. Therefore, these are individuals who are likely to undergo WB-MRI scans in the near future due to financial reasons, and our findings are highly relevant in this context. However, the present study also has some limitations.

The major limitation of our study is the relatively low number of subjects, although it is comparable to other previous investigations.^9^ In addition, follow-up analysis of disclosed potentially relevant incidental findings is still incomplete.

In conclusion, our data suggest that 3 T wide bore WB-MRI, DWI and MRA of high diagnostic accuracy lead to the detection of clinically relevant diseases and many incidental findings in a cohort of healthy adults that require further imaging or surveillance in two-third of subjects. Furthermore, research involving large numbers of patients is required to determine the potential benefit or burden of communicating incidental findings to study volunteers. Our research was the first one which involved this highly selected patient group, using a high field 3 T wide bore magnet system with T1, STIR, whole-body DWI and MRA acquisitions. Our study was the first which added a DWI sequence to the WB-MRI screening protocol which might help the depiction of areas of malignancies.

## Figures and Tables

**TABLE 1.** Spatial resolution data of our study acquisitions

WB-T1	448 × 256	voxel	Freq. 1,12
			Phase 1,95
			Sl. 5
WB-STIR	384 × 224	voxel	Freq. 1,3
			Phase 2,23
			Sl. 5
WB-DWI	128 × 128		Freq. 3,9
			Phase 3,9
			Sl. 8
WB-MRA – Thorax/Abdomen/Pelvis	288 × 192		Freq. 1,67
			Phase 2,5
			Sl. 2,8
WB-MRA – Upper extremity	384 × 192		Freq. 1,25
			Phase 2,5
			Sl. 2,4
WB-MRA – Lower extremity	320 × 192		Freq. 1,5
			Phase 2,5
			Sl. 1,8

WB = Whole-body; STIR = Short TI Inversion Recovery; DWI = diffusion weighted imaging; MRA = magnetic resonance angiography, Freq.: frequency, Sl.: slice thickness in mm
